# Role of Arachidonic Acid and Its Metabolites in the Biological and Clinical Manifestations of Idiopathic Nephrotic Syndrome

**DOI:** 10.3390/ijms22115452

**Published:** 2021-05-21

**Authors:** Stefano Turolo, Alberto Edefonti, Alessandra Mazzocchi, Marie Louise Syren, William Morello, Carlo Agostoni, Giovanni Montini

**Affiliations:** 1 Fondazione IRCCS Ca’ Granda-Ospedale Maggiore Policlinico, Pediatric Nephrology, Dialysis and Transplant Unit, Via della Commenda 9, 20122 Milan, Italy; aedefonti@hotmail.com (A.E.); william.morello@policlinico.mi.it (W.M.); giovanni.montini@policlinico.mi.it (G.M.); 2 Department of Clinical Sciences and Community Health, University of Milan, 20122 Milan, Italy; alessandra.mazzocchi@unimi.it (A.M.); eva.syren@unimi.it (M.L.S.); carlo.agostoni@unimi.it (C.A.); 3 Fondazione IRCCS Ca’ Granda Ospedale Maggiore Policlinico, Pediatric Intermediate Care Unit, 20122 Milan, Italy

**Keywords:** kidney, arachidonic acid, nephrotic syndrome

## Abstract

Studies concerning the role of arachidonic acid (AA) and its metabolites in kidney disease are scarce, and this applies in particular to idiopathic nephrotic syndrome (INS). INS is one of the most frequent glomerular diseases in childhood; it is characterized by T-lymphocyte dysfunction, alterations of pro- and anti-coagulant factor levels, and increased platelet count and aggregation, leading to thrombophilia. AA and its metabolites are involved in several biological processes. Herein, we describe the main fields where they may play a significant role, particularly as it pertains to their effects on the kidney and the mechanisms underlying INS. AA and its metabolites influence cell membrane fluidity and permeability, modulate platelet activity and coagulation, regulate lymphocyte activity and inflammation, preserve the permeability of the glomerular barrier, influence podocyte physiology, and play a role in renal fibrosis. We also provide suggestions regarding dietary measures that are able to prevent an imbalance between arachidonic acid and its parental compound linoleic acid, in order to counteract the inflammatory state which characterizes numerous kidney diseases. On this basis, studies of AA in kidney disease appear as an important field to explore, with possible relevant results at the biological, dietary, and pharmacological level, in the final perspective for AA to modulate INS clinical manifestations.

## 1. Introduction

Idiopathic nephrotic syndrome (INS) is one of the most frequent glomerular diseases in childhood [1]. It is characterized by proteinuria, caused by podocyte damage, hypoalbuminemia, hyperlipidemia, and edema [1]. While the exact cause of podocyte damage is still not completely understood [1], it is well known that hyperlipidemia is related to urinary loss of transport proteins, which carry free cholesterol, and to the consequent compensatory increase in the synthesis of proteins involved in triglyceride metabolism [1]. Two theories have been proposed to explain the pathogenesis of edema in INS. According to the classical underfill hypothesis, hypoalbuminemia reduces plasma oncotic pressure, which leads to sodium and water retention and water leakage into the interstitium [2]. Meanwhile, the overfill hypothesis postulates proteinuria to be the primary cause of sodium retention, with consequent volume expansion and leakage of excess fluid into the interstitium [3].

Other biochemical alterations were also described in INS, such as changes in pro- and anti-coagulation factors’ levels and increased platelet count and aggregation, leading to a hypercoagulable state [4].

Based on their response to corticosteroid therapy, children with INS are classified as steroid-sensitive patients, which includes those with infrequent relapses, frequently relapsing or steroid-dependent patients who present a favorable prognosis, or steroid-resistant patients, who carry an unfavorable prognosis in the majority of cases. Histopathology usually reveals minimal change of disease, which is characterized by normal glomerular appearance on light microscopy and evidence of podocyte foot processes’ alterations on electron microscopy; focal segmental glomerulosclerosis and interstitial fibrosis may be found in steroid-resistant cases [5,6].

The pathogenesis of INS has not yet been fully clarified. Excluding genetic causes, the main theory for immune-mediated cases involves a dysfunction of T lymphocytes, which would switch to the production of still poorly defined permeability factors that interfere with the expression and/or function of key proteins in the podocyte, thus being the main culprits of proteinuria [7]. Candidates for the circulating factors that affect glomerular permeability include angiopoietin-like 4 (ANGPTL4), cardiotrophin-like cytokine-1 (CLC-1), and soluble urokinase plasminogen activator receptor (suPAR) [1].

Arachidonic acid (AA) is a long-chain polyunsaturated fatty acid of the omega-6 group and represents 7% to 10% of total circulating fatty acids; it is the second most abundant omega-6 fatty acid in the human body [8] (Table 1), with linoleic acid (LA) being the first. AA is synthesized endogenously from LA through three steps mediated by two enzymes, desaturase and elongase, and may also be derived from the diet. In turn, AA is a substrate of elongases for the synthesis of longer fatty acids of the omega-6 series. 

AA is metabolized by three types of oxygenases: cyclooxygenase (COX), lipoxygenase (LOX), and cytochrome P450, leading to the generation of eicosanoids, namely prostaglandins, thromboxane, leukotrienes, and hydroxyeicosatetraenoic acids.

**Figure 1:**
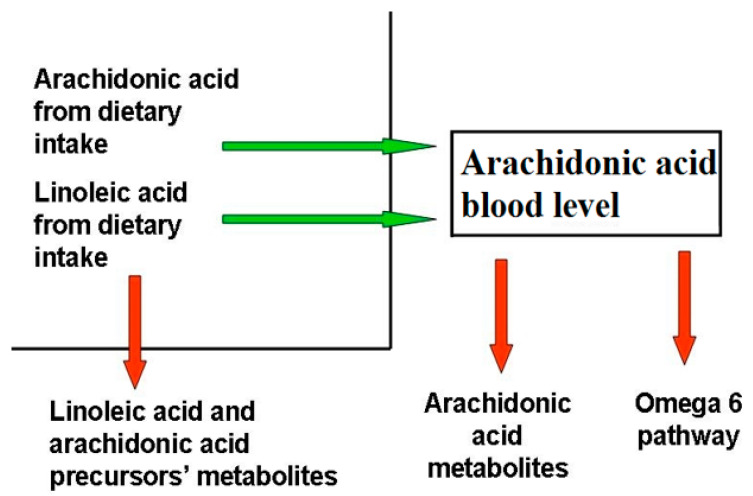
Factors determining blood arachidonic acid levels. The factors influencing AA levels belong to two categories, those that increase (green arrow) AA blood levels, like diet uptake and the omega-6 pathway from LA, and those that decrease them (red arrow), like the synthesis of omega-6 FA downstream AA, AA metabolization, and the reduction of AA precursors due to their metabolization. Diseases able to modify AA blood levels act through the same mechanisms.

Blood AA levels do not reflect its synthesis and metabolization pathways (Figure 1), as they are maintained as constant, even at the expense of other biological factors, as observed in patients with epidermolysis bullosa [9], where, despite the large amount of active AA metabolites, the AA level is comparable to that of healthy controls. This phenomenon has been observed in several other chronic inflammatory disorders, for instance cystic fibrosis [10], even if the exact mechanism behind it is unclear.

AA is involved in several biological processes, either in health or disease. Herein, we describe its role in nephrotic syndrome from a biological and clinical perspective. AA influences cell membrane fluidity and permeability and modulates platelet function and immune system activation; furthermore, it affects glomerular and tubular function, the physiopathology of podocyte, and the process of renal fibrosis. We also detail the interactions between AA and the common drugs prescribed for INS treatment. Finally, the role of dietary AA balance and its nutritional sources are discussed.

For this review, PubMed (www.pubmed.gov, accessed on 28 February 2021) was the only source of the articles. No limit was given regarding the date of publication of the articles, and the following keywords were used: arachidonic acid, arachidonic acid metabolism, cell membrane, immune system, nephrotic syndrome, membrane receptor, coagulation, platelets, arachidonic acid pathway, TXA_2_, LTB_4_, PGE_2_, CNI pharmacogenomics, cyclosporine A, tacrolimus, kidney disease, arachidonic acid and kidney, podocyte, podocyte and arachidonic acid, 20-HETE, 20-HETE metabolism, renal fibrosis, SNI pharmacogenomics, CYP, and all the key words related to the biological mechanism reported in each chapter.

## 2. Cell Membrane Fluidity and Permeability

It was recently described that erythrocyte membranes of patients with INS differ from those of normal subjects, particularly due to reduced membrane fluidity [11].

AA is one of the most abundant fatty acids in the cell membrane, to which it endows mobility and flexibility [12,13]. The fatty acid composition determines the viscosity of the cell lipid bilayer and membrane fluidity, thus directly affecting the function of specific membrane proteins, like, for example, those involved in cellular inflammatory signaling, namely lymphocyte function-associated antigen 1 (LFA-1), intercellular adhesion molecule 1 (ICAM-1), and cluster of differentiation 2 (CD2) [12,13]. 

With regard to membrane permeability, AA acts on Ca^2+^ cell load [10] with a double effect: at low micromolar concentrations it increases Ca^2+^-ATPase activity, while at higher concentrations it reduces ATPase activity. This may be due to an unspecific and non-physiological inhibitory effect on the hydrolytic activity of P-type ATPase. ATPases are a superfamily of lipid pumps involved, among other functions, in secretion and absorption at the kidney level; these pumps are blocked by protein kinase C inhibitors [14]. AA increases membrane permeability to calcium, which is a key factor for platelet activation [15].

AA may act on ion channels by either binding to or inserting among the membrane molecules, thus modifying the mechanical properties of the cell membrane and modulating channel function [16].

AA also has a direct effect on several membrane potassium channels, either by accelerating their inactivation (in particular, the A-type channels and delayed rectifier channels), or by inducing the activation of large-conductance voltage-independent channels. The two-pore domain potassium channels are inactivated by AA as well, in contrast to what usually occurs with classical K channel-blocking drugs [16]. Transient receptor potential channels (TPR) are instead activated directly by AA and its lipoxygenase (LOX)-derived metabolites [16] (namely, 12- and 15-(*S*)-hydroperoxyeicosatetraenoic acids, 5- and 15-(*S*)-hydroxyeicosatetraenoic acids, and leukotriene B_4_). LOX metabolites can activate the TPR channel by virtue of their structure that mimics the capsaicin structure [17]. Interestingly, AA and its metabolic byproducts effects on calcium and potassium balance at the membrane level have been hypothesized to underlie the molecular-related derangements in INS [6].

As it concerns membrane fluidity, albumin is the main fatty acid-binding protein in extracellular fluid, having seven fatty acid-binding sites [18]. Albumin increases AA release from cell membranes in a concentration-dependent manner, by interacting with membrane phospholipids on the extracellular surface; in particular, positively charged arginine residues at or near albumin’s binding sites for LCFA interact with AA, determining its release from the phospholipid layer [19]. Thus, albumin decreases cell membrane permeability of endothelial and circulating cells to water and small solutes [19]. 

In conclusion, the amount of AA in cell membranes regulates several cellular functions and all factors that vary the amount of AA in the membrane may play a significant pathogenetic role in renal disease.

## 3. Platelet Aggregation and Coagulation 

**Figure 2:**
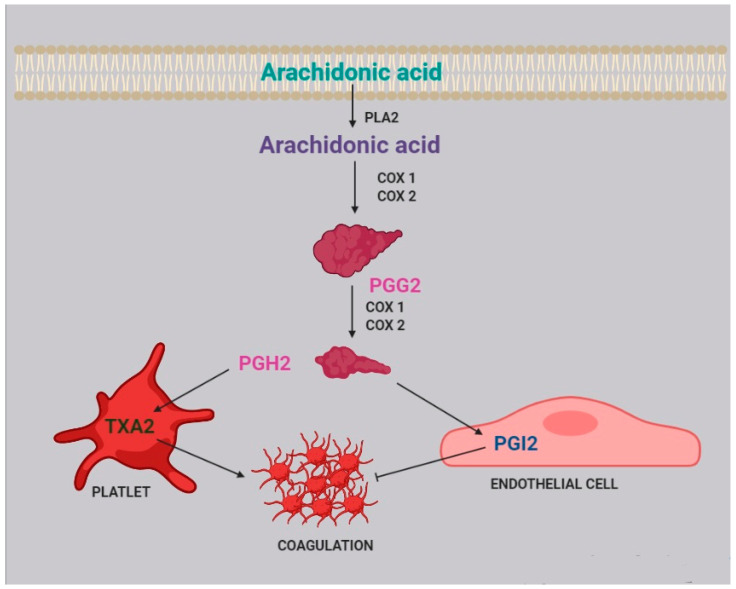
Role of arachidonic acid in the coagulation process. Arachidonic acid is released from cell membranes by phospholipase A_2_ (PLA_2_) and subsequently metabolized by COX1 and COX2 to obtain prostaglandin G_2_ (PGG_2_) and H_2_ (PGH_2_).

Two of the most active compounds related to platelet function are thromboxane and prostacyclin, both metabolites of AA [20]. AA is released from platelet membrane by phospholipase A_2_ (PLA_2_), which hydrolyzes the bond between the second fatty acid of phospholipids and the glycerol molecule. The released AA is then metabolized by cyclooxygenase [21], generating prostaglandin G_2_, and thereafter prostaglandin H_2_. Afterwards, two different pathways can take place: the first one, within the platelets, leads to the synthesis of thromboxane A_2_ (TXA_2_) and subsequently B_2_ (TXB_2_); the second one, within the endothelial cells, leads to the synthesis of prostacyclin (PGI_2_) (Figure 2).

TXA_2_ stimulates platelet activation and aggregation, via platelet fibrinogen-binding αIIbβ_3_ receptors [4]. Prostacyclins, in contrast, inhibit platelet activation by activating G protein-coupled receptors on platelets and endothelial cells. Upon binding to the prostacyclin receptor, PGI_2_ induces adenyl cyclase cAMP production, which in turn inhibits platelet activation [22].

A higher incidence of increased platelet aggregation and thromboembolism has been reported in nephrotic syndrome, in relation to consistently elevated levels of fibrinogen. Moreover, both hyperlipidemia and hypoalbuminemia, which are characteristic findings of nephrotic syndrome, increase thromboxane availability, through the production of TXA_2_ precursors and the removal of TXA_2_ inhibitors [23]. The exact mechanism underlying this process is still unknown, but it probably involves an increase in PLA_2_ activity, related to the abnormally high cholesterol levels [24]. Therefore, arachidonic acid, which is the precursor of thromboxane, may be considered a crucial player in the platelet-related coagulation process.

It is worth noting that in a clinical trial that recruited six healthy male volunteers, fed for 50 days with a diet containing 1.7 gr/day of AA, and six controls, fed with a diet containing 210 mg/day of AA [20], moderate intakes of foods rich in AA, like those of the first group, had only mild effects on blood coagulation, platelet function, and platelet fatty acid composition compared to controls. The authors attributed the poor efficacy of arachidonic acid supplementation to the moderate amount in which it was supplied. 

In platelets, PGH_2_ is metabolized to tromboxane A_2_, activating coagulation and platelet aggregation, while in endothelial cells, prostaglandin I_2_ (PGI_2_), which has an anticoagulant effect, is generated.

## 4. Immune System

The therapeutic efficacy of Rituximab in modifying the course of steroid-dependent nephrotic syndrome suggested that B cells play a key role in the pathogenesis of INS. This was recently confirmed by evidence of a pathological increase in memory B cells in INS [25]. Moreover, other studies showed a decrease of Treg cells [26], dysregulation of T-cells [27], lower levels of NK and NKT cells, and increased levels of inflammatory markers during proteinuria [28,29]. These studies confirm [1] that the immune system plays a pivotal role in non-genetic INS and specifically in the loss of the glomerular barrier function, by activating the inflammatory process against podocytes.

In immune cells, like lymphocytes, neutrophils, and monocytes, AA constitutes about 20% of total fatty acids, while EPA and DHA constitute 1% and 2.5%, respectively [30]. It was reported that oral administration of omega-3 fatty acids changes the pattern of production of eicosanoids, by increasing resolvins production, thus affecting phagocytosis, T-cell signaling, and antigen presentation capability. These effects seem to be mediated at the membrane level [30].

The distribution of AA within intracellular lipid pools in inflammatory cells has an important role in regulating eicosanoids production. In fact, a pool of AA was identified within the triglycerides of mast cells, eosinophils, monocytes, and platelets [31].

When inflammatory cells are activated, AA is released from membrane phospholipids into the cell and partially incorporated into intracellular triglycerides, ready to supply membrane phospholipids again after cell activation has ended [32].

Thus, AA metabolites can act in several ways on lymphocyte activity, affecting inflammation levels [32,33,34] (Table 2) and possibly the course of INS. 

Regarding B, NK, and T cells, the main AA metabolites involved are PGE_2_, LTB_4_, and TXA_2_.

PGE_2_ is produced by nearly all cells within the body [35]. Secreted PGE_2_ acts in an autocrine or paracrine manner through its four cognate G protein-coupled receptors EP1 to EP4 [36]. It inhibits T-cell and NK-cell proliferation, as well as IFN-γ and IL-12 production [37], binding their cell-surface receptors [38]. PGE_2_ also inhibits B-cell activation secondary to IL-4 stimulation in a specific manner and enhances IgE and IgG1 production [39].

LTB_4_ exerts pleiotropic effects on lymphocytes and regulates the immune response in a dynamic, cell type- and context-dependent manner: LTB_4_ enhances T-cell recruitment, it inhibits de novo iTreg generation and increases interleukin-17 (IL-17) cytokine production during T-cell differentiation. LTB_4_ also regulates the migration of various lymphoid-derived cell types in different ways that vary depending on disease and tissue. [40].

TXA_2_, another product of AA metabolism, inhibits naïve T-cell proliferation and exerts several effects on mature T lymphocytes: it inhibits T-cell interaction with dendritic cells, increases T-cell proliferation and activation, and has been shown to topically enhance the cytotoxic activity of immune cells [37].

Moreover, eosinophils, mast cells, macrophages, dendritic cells, and Th2 lymphocytes have surface membrane receptors for arachidonic-derived metabolites, in particular for prostaglandin D_2_, cysteinyl leukotrienes D_4_ and E_4_, and lipoxin A_4_ [33], but these findings have not been confirmed so far in patients with INS.

A pharmacological modulation of AA metabolites could decrease the inflammatory damage to the podocyte. The pathogenetic role of AA is supported by the fact that medications have been recently administered to target AA metabolism and decrease kidney inflammation [21,34]. They include aspirin, nimesulide, licofelone, baicalein, and others. Some of them are in the early stages of development for kidney diseases like diabetic nephropathy, glomerulonephritis, and idiopathic membranous nephropathy [34].

## 5. Kidney Glomerular and Tubular Function

Epoxyeicosatrienoic acids (EETs) are produced in several tissues, like the heart, the muscles, the kidneys, the pancreas, the lungs, and the brain [41], but mainly in the vascular endothelium, in response to various PLA_2_-activating stimuli, EETs activity could be reduced by metabolization made by soluble epoxyde hydrolase (sHE) [42].

EETs modulate kidney function acting directly on tubular ionic transport, vascular tone, and cellular proliferation, and have a nephro-protective role [43] due to their anti-inflammatory properties.

EETs in fact induce vasodilatation in an autocrine manner [44] and have anti-apoptotic activity; it was also reported that their generation is reduced in case of renal disease [42], even if no explanation regarding such a mechanism was given.

Glomerular inflammation is mitigated by EETs, which decrease the influx of neutrophils and macrophages and decrease the production of cytokines, monocyte chemotactic protein-1, TNF-α, macrophage inflammatory protein 2, and ICAM-1 [45]. The protective effect is due to EETs renal vasodilator and antipressor response to salt loading, through the inhibition of renal tubular Na^+^ reabsorption and the increase of Na^+^ renal excretion, resulting in an anti-hypertensive effect [46] that is probably mediated by A2A receptors [47].

20-HETE, another eicosanoid derived from AA metabolism, shares the same protective properties as EETs [48]. It plays a predominant role in the regulation of renal tubular and vascular function, and variants in the genes encoding for the enzymes that produce 20-HETE are associated with hypertension [49].

It has been shown that sustained production of 20-HETE in the glomerulus is required to maintain the glomerular permeability barrier to albumin [48]. It is still unclear which cell types in the glomerulus express the CYP enzymes that synthetize 20-HETE, and the exact mechanisms by which this molecule influences the glomerular permeability barrier are yet to be defined as well [48], although it is likely that its effects are mediated by the modulation of Na^+^-K^+^-ATPase, Na^+^-K^+^- 2Cl^−^ cotransporter, and K^+^ channel activity in nephrons [49] through the activation of the PKC pathway [50].

20-HETE is also involved in podocyte apoptosis, by regulating the canonical transient receptor potential-6 (TRPC6) channels and increasing the Ca^2+^ flux [51].

In patients affected by nephrotic syndrome who develop early hypertension [52], a decreased concentration of 20-HETE in the proximal tubule has been observed [53] in association with increased albumin permeability in the glomeruli, which worsens proteinuria and glomerular injury [54]; this finding supports the role of 20-HETE in preserving glomerular permeability barrier to albumin. However, it is unknown if the reduction of 20-HETE causes or is caused by hypertension.

In conclusion, 20-HETE may act in different ways (protective or pro-apoptotic) in different cell types and kidney regions.

## 6. Podocyte Physiopathology and Infections

It is well known that, in the course of nephrotic syndrome, infections lead to an exacerbation of proteinuria [55] and are an important risk factor for relapses [1,56,57].

Infections activate the immune system, triggering the inflammatory cascade. It was recently reported that during inflammation two enzymes are induced: 15-lipoxygenase (15-LO) and secreted phospholipase A_2_ (sPLA_2_) [58]. Notably, 15-LO is expressed in human podocytes [59], while sPLA_2_ is expressed in platelets, neutrophils, eosinophils, and macrophages [60]. sPLA2 releases AA from membrane phospholipids [51,61] acting in a paracrine way.

In glomerular podocytes, intracellular free AA is metabolized to PGE_2_, which, by interacting with the EP 4 receptor (prostaglandin E_2_ receptor 4) expressed by podocytes, reduces AA release [52]. This loop regulates podocyte function in both physiological and pathological conditions and is able to change PGE_2_ synthesis [62].

As described above, during the course of an infection, intracellular AA levels increase due to the action of sPLA_2._ It was reported that in podocytes, an excess of AA activates protein kinase A, which in turn promotes c-Abl activation and nephrin phosphorylation, thus causing actin cytoskeleton remodeling and podocyte injury [63]. This mechanism could partially explain the frequent recurrence of proteinuria during infectious episodes in children. Moreover, an increase in sPLA_2_ 1B levels and PLA_2_R expression has been observed to be positively associated to podocyte apoptosis in kidneys of patients with idiopathic membranous nephropathy [64].

Podocyte foot process injury and podocyte apoptosis due to cytoskeleton remodeling were also attributed to a change of Ca^2+^ efflux [51], driven by 20-HETE, the main AA metabolite. It has also been observed that 20-HETE increases the current Ca^2+^ flowing through TRPC6 channels in the podocyte [51], which are located at the slit diaphragm, possibly leading to cellular injury.

Studies on the relation between AA and the podocyte are scant, but very promising for increasing our knowledge of the pathogenesis of renal damage in INS.

## 7. Renal Fibrosis

Renal fibrosis is a process that progresses independently of the primary renal disease [65] and represents a failed wound-healing process of the kidney tissue. Renal biopsies of patients with steroid-resistant nephrotic syndrome often show glomerulosclerosis and interstitial fibrosis, which are associated with progression to end-stage kidney disease in more than 50% of cases [65], a poor prognosis that heightens the necessity of increasing our knowledge of the mechanisms underlying fibrosis.

Renal fibrosis is characterized by connective tissue deposition in the kidney parenchyma, particularly in the interstitial space and within the walls of glomerular capillaries, and by the consequent cellular processes. Fibrosis also interferes with normal tubular function, leading progressively to organ failure [65,66].

The scar tissue contains fibrillar collagen I and III as well as some constituents of the normal capillary basement membrane, like collagen IV and V, fibronectin, laminin, perlecan, and heparin [66].

Fibrosis is associated with leukocyte recruitment, angiogenesis, vascular leak, and the appearance of myofibroblasts. In particular, both the glomerulus and the interstitium attract large numbers of leukocytes, the majority of which are of myeloid lineage, and mostly neutrophiles in acute settings, whereas macrophages and dendritic cells predominate in chronic settings. In the case of chronic immune-mediated diseases, T lymphocytes are predominant [66].

Activated macrophages may either damage the tissue directly or generate profibrotic cytokines, including TGF-β and other growth factors, and are capable of producing some matrix constituents. It is therefore evident that fibrosis and renal inflammation, primarily driven by immune system activation, are closely related.

Beyond its role in immune function regulation, AA is also directly related to fibrosis. In vitro experiments of cell cultures incubated with PUFAs showed that AA is able to induce upregulation of the expression of TGF-β, fibronectin 1 (FN1), connective tissue growth factor (CTGF), and collagen IV, all compounds related to fibrosis [67]. AA also enhances in vitro angiotensin II (AngII)-induced gene expression [67], activating mechanisms that mediate renal damage. Interestingly, omega-3 EPA and DHA, if administered with AA, suppress the effects of both AA and AngII [67]. On the other side, angiotensin II is degraded to form angiotensin-(1-7), which inhibits angiotensin II-stimulated phosphorylation of the mitogen-activated protein kinases (MAPKs) p38, extracellular signal-related kinase (ERK1/ERK2), and C-JUN N-terminal kinase (JNK) in proximal tubular cells, thus exerting a protective role against fibrosis. As a matter of fact, the p38 MAPK phosphorylation leads to the release of AA and the production of TGF-β 1 and extracellular matrix proteins [68].

20-HETE, an AA metabolite, also plays a distinct role in fibrogenesis, by activating the renin-angiotensin-aldosterone system (RAAS), by inducing vascular expression of ACE downstream of NF-κB activation [69,70]. It is well known that the RAAS is involved in renal fibrosis [71], because it increases TGF-β expression, which starts a biomolecular cascade driving to renal fibrosis.

On the contrary, PGE_2_, another AA metabolite, has been shown to inhibit collagen type 1 production and to induce matrix metalloproteinase 1 (MMP1) expression in dermal fibroblasts [72] by binding to the EP-1 receptor on fibroblasts, starting a pathway-regulated ERK1/2 and IP3 signaling that leads to a reduction in collagen expression and an increase in MMP1 expression [72].

In summary, AA and its metabolites play a significant role in the main mechanisms responsible for irreversible renal damage, within a complex network where the final result may be variable, according to genetic- and environment-associated factors. The metabolic balance between mediators of the intermediate metabolism, more than the individual compounds, may dictate the final effects. Moreover, it is still uncertain if the final, relevant pathologic effect, that is, renal fibrosis, could be regulated by dietary or pharmacological measures addressed to directly modify blood AA levels or indirectly modify the activity of AA metabolites.

## 8. Drug and Gene Interactions

Idiopathic nephrotic syndrome is usually treated with glucocorticoids or with immunosuppressive drugs, particularly calcineurine inhibitors (CNI), such as cyclosporine A (CsA) and tacrolimus (Fk). CNIs are metabolized mainly by cytochrome P450, encoded by the CYP gene cluster. As seen above, the CYP gene is also involved in AA metabolism, but in the literature, there are no reports of enzymatic competition between these drugs and AA.

With regard to the relationship between CNI and AA blood levels, an in vitro study reported that CsA decreases the activity of Delta 9 desaturase and increases the activity of Delta 6 and Delta 5 desaturases [73] through unknown mechanisms. However, as Delta 5 desaturase is involved in the last step of AA biosynthesis [73], CsA therapy could increase AA blood level of patients with INS.

On the same line, it has been suggested that CsA mostly increased the availability of free AA instead of decreasing AA blood levels through the acceleration of AA conversion by the cyclooxygenase pathway [74], but a further in vitro study concluded that CsA had no effect on AA release and metabolism [75]. This result was confirmed more recently in a study of CsA and glucocorticosteroids in human peripheral blood mononuclear cells [76].

With regard to the role of AA metabolism in determining CNI side effects, it is well known that CsA treatment may cause gingival overgrowth, which depends on PGE_2_ production in gingival fibroblasts. In fact, CsA potentiates TNF-α to stimulate the release of AA from fibroblasts, with consequent enhanced production of PGE_2_ and gingival overgrowth [77]. There are no studies reporting the same effect in other tissues.

The nephrotoxicity of CsA is well established, and Fk administration is associated with the same side effect, which has been linked to CYP2C8*3 and CYP2C8*4 polymorphisms and a consequent reduction of EETs: it was observed that a circulating Fk plasma concentration of 10 ng/mL is able to reduce the production of eicosanoids by 35%. It follows that CNIs-induced nephrotoxicity could be due to a reduced activity of CYP2C8*3, which reduces the production of EETs, enhancing drug nephrotoxicity [78].

Pre-treatment with Fk is also known to enhance glucocorticoids to inhibit AA and PGE_2_ production [79] by inhibiting COX2 expression, but the co-administration of Fk and glucocorticoids does not inhibit COX2 expression, allowing for normal PGE_2_ production [79].

Therefore, the main drugs that are commonly used in INS may have several effects on AA metabolism, which are possibly related to an increased utilization. While contrasting findings have been reported, some of these may account for some drug side effects, like gingival overgrowth and nephrotoxicity, and even therapeutical actions. Although the incorporation of omega 3 as an oil excipient to CsA, to increase bioavailability and decrease nephrotoxicity, has been investigated with interesting results [80,81], no practical therapeutic strategies have been developed so far.

## 9. Dietary Balance Between AA and LA and AA Sources

AA, which belongs to the omega-6 series, and docosahexaenoic acid (DHA), which belongs to the omega-3 series, are the most important byproducts of essential fatty acids linoleic and α-linolenic acid, and their imbalance has been associated with inflammatory and chronic disorders [82].

While LA and AA are mostly known as inflammatory molecules, operating within an interdependent network through their metabolites [82], AA metabolites also have anti-inflammatory and protective roles, while LA metabolites affect immune function by binding cellular receptors and altering signaling molecules [83].

AA and DHA levels depend on both to genetic predisposition and diet intake. Blood AA levels, as shown in Figure 1, can be modulated through dietary habits, taking into account that there is a marked difference between the amount of AA supplied with the diet and the amount synthesized by human metabolic pathways. In the latter case, the main rate-limiting enzymes are the Δ5- and Δ6-desaturases, which are encoded by the genes FADS1 and FADS2, and different polymorphisms in the fatty acid desaturases genes might even increase or decrease the production of these LC-PUFAs [84]. As a matter of fact, looking at the frequencies of the 28 SNPs in the FADS haplotypes, their distribution in the 3 main haplotypes is evident over the world [85].

Unlike other fatty acids, omega-3 and omega-6 precursors (LA and linolenic acid, respectively) cannot be synthesized de novo by mammals (they are essential dietary compounds indeed), so the relative abundance of these PUFAs in the diet has a major influence in humans.

LA is the most represented omega-6 PUFA in most western diets, and is widely distributed in foods: it represents more than 50% of the lipid content in various vegetable oils, including safflower, sunflower, corn, and soybean oils; it is present in high amounts in nuts and seeds, while lower levels are found in whole grains, legumes, some meats, eggs, and dairy products [86]. Notably, it was recently reported that a strong reduction in dietary intake of LA was not associated with a linear decrease in circulating AA levels [87].

The AA state depends on the endogenous synthesis from the essential precursor LA, undergoing desaturation and elongation, and the direct dietary intake [88]. Since LA to AA conversion efficiency is low in humans, AA intake through the diet appears to be significantly more effective in raising its circulating levels.

In contrast to LA, AA is relatively scarce in the diet and is found in meat (both red and white, including fish), organ meats (e.g., liver, kidney, brain), and eggs, with minimal amounts in cow’s milk fat and the products derived from it [89]. The mandatory origin from animal sources is directly connected to the ability of animals to derive it through enzymatic activities, acting on the vegetable essential precursor, LA. In particular, animal sources are the most representative. Diets rich in meat of beef, lamb, pork, and poultry are claimed to contribute to the high tissue AA content [90]. This amount is influenced by the diet composition, the digestive system, and the biosynthetic processes within the animal [91].

Macroalgae, fungi, bacteria, and yeasts can be a source of essential PUFAs, which may provide humans with fatty acids when included in the diet or used as feed for fish and livestock.

Many fungi, yeast, and some bacteria can synthesize great amounts of LC-PUFAs, mostly AA. The most efficient AA-producer fungus is the non-pathogenic *Mortierella* spp., in which AA production accounts for up to 70% of total lipids [92].

Among algae, AA has been identified in many groups that grow photoautotrophically or heterotrophically. Certain algal species are reported to contain a naturally higher AA content, which may reach 77% of total fatty acids, as in freshwater green microalga *Parietochloris incisa*, 40% of total fatty acids in the red alga *Porphyridium purpureum,* and 20–30% in diatoms such as *Phaeodactylum tricornutum* and *Thalassiosira pseudonona*. AA has been detected in lesser amounts in some lichen species (symbiosis association between fungi and algae). Lower plants, such as mosses and ferns, have higher amounts of AA than seagrasses and terrestrial higher plants [92].

Over the last century, dietary intake of n-3 LC-PUFA has decreased, while the dietary content of LA has increased, driven by the use of vegetable products that are rich in LA, and industrialized foods produced with LA-rich vegetable sources [83,93].

From a therapeutic point of view, the achievement of a balance between individual polymorphisms in FADS genes modulation and PUFAs dietary intake could improve or prevent inflammatory conditions, especially in those subjects who can take advantage from an exogenous dietary PUFA supply, due to poorer endogenous synthesis rates [93]. The Mediterranean diet, which is balanced in omega-3 fatty acids from vegetal fats and fish, gets few AA through limited amounts of animal products, and is poor in LA, appears today as an optimal reference for dietary pattern as it concerns the prevention of inflammatory states. Indeed, a working hypothesis to be tested indicates that a reduction of LA amounts in diet, together with a balanced AA intake from natural sources, could be a further way to reduce the inflammatory potential of diets, and in parallel to increase of the supply of n-3 fatty acids.

## 10. Conclusions

**Figure 3:**
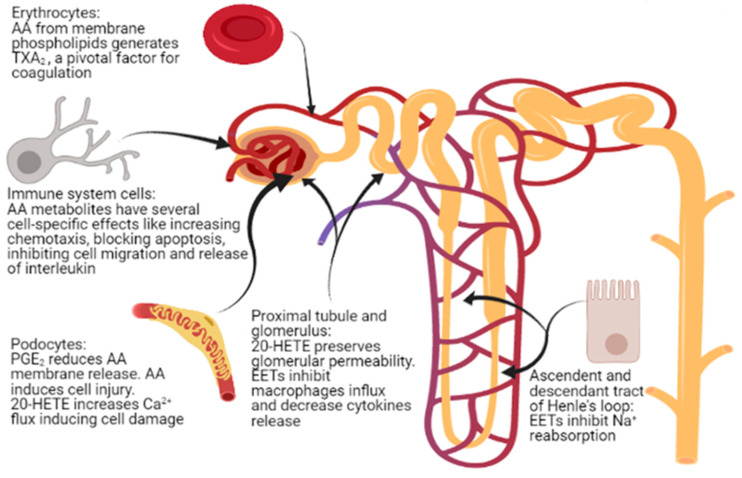
Sites of action of arachidonic acid (AA) and its metabolites in nephrotic syndrome.

AA and its metabolites play multiple roles, influencing the structure and function of cells such as platelets, lymphocytes, and podocytes (Figure 3), and thus being involved in processes such as coagulation, inflammation, and fibrosis, as well as in maintaining the integrity of the glomerular basement membrane. However, studies concerning the role of AA in kidney disease, including INS, are scarce. Pharmacological and dietary interventions capable of modulating AA and its metabolites are under study, but no clinical trials regarding the role of a diet rich in PUFAs in patients with INS have been published so far. Moreover, a correct dietary balance between AA and LA may represent a further relevant anti-inflammatory measure that should be tested in a controlled context. Accordingly, studies of AA and its metabolites seem an important field to explore, particularly in idiopathic nephrotic syndrome, with possible relevant consequences at the biological, dietary, and pharmacological levels, with the final perspective of obtaining an effective modulation of AA endogenous metabolism, finally counteracting some pathogenetic mechanisms of kidney damage.

## Figures and Tables

**Table 1:** Blood omega-6 levels in healthy subjects. Linoleic acid, AA, omega 6, total saturated fatty acid, monounsaturated fatty acids, and total omega-3 levels in human subjects. AA is the second highest fatty acid of the omega-6 series. Data are expressed as percentage of total fatty acids [4].

	Neonates	Children	Adults	Elderly
Linoleic acid (%)	4.61 ± 1.06	17.67 ± 1.92	18.41 ± 2.87	17.64 ± 2.89
Arachidonic acid (%)	13.14 ± 1.73	8.33 ± 1.04	8.51 ± 1.38	8.32 ± 1.40
Total omega 6 (%)	22.99 ± 2.13	28.97 ± 2.19	29.79 ± 3.13	28.78 ± 3.24
Total saturated fatty acids (%)	46.10 ± 3.16	44.32 ± 1.61	39.47 ± 2.3	39.83 ± 2.16
Total monounsaturated fatty acids (%)	26.15 ± 2.76	24.39 ± 2.07	27.20 ± 3.08	27.83 ± 3.27
Total omega 3 (%)	4.76 ± 0.89	2.31 ± 0.50	3.54 ± 1.05	3.55 ± 0.95

**Table 2:** Effects of AA metabolites on immune cells [38]; AA metabolites affect immune cells in various ways, modulating the immune response and inflammation.

Cell Type	AA Metabolite	Effect
Basophil	PGD_2_	Stimulates basophil chemotaxis
Eosinophil	PGD_2_	Stimulates eosinophil chemotaxis
		Blocks eosinophil apoptosis
		Activates eosinophils
Naive t cell	TXA_2_	Inhibits proliferation of naive T cells
B-cell	PGE_2_	Enhances IgE class switching by B cells
Dendritic cell	LTB_4_	Stimulates DC production of IL-6
	LTC_4_	Participates in cell migration
		Enhances cells activation and functions
	PGD_2_	Inhibits cells migration
	PGE_2_	Stimulates IL-10 production
		Modulates cell migration
		Downregulates major histocompatibility complex C class II expression
		Inhibits IL-12 and IFN-ã production
		Inhibits the expression of CCL3/CCL4
	PGJ_2_	Induces apoptosis
	TXA_2_	Inhibits interaction with T cell
Langerhans cell	PGE_2_	Promotes the migration and maturation of Langerhans cells
	PGD_2_	Inhibits cells migration
Lymphocyte	PGE_2_	Inhibits interactions with endothelial cell
Macrophage	PGE_2_	Suppresses cytokine production
		Suppresses chemokine expression
	PGJ_2_	Inhibits release of IL-10 and IL-12
Mast cell	PGE_2_	Enhances antigen-stimulated degranulation
Neutrophil	LTB_4_	Activates cells
NK cell	PGE_2_	Inhibits IL-12 and IFN-ã production
T cell	LTB_4_	Enhances cell recruitment
	PGE_2_	Inhibits cell proliferation
	TXA2	Inhibits interactions with dendritic cells
		Regulates the elimination of self-reactive cells
		Increases cell proliferation and activation
		Enhances local cytotoxic cell function
Th2	PGD_2_	Stimulates chemotaxis
